# Utilization Pattern of Traditional Chinese Medicine among Fracture Patients: A Taiwan Hospital-Based Cross-Sectional Study

**DOI:** 10.1155/2018/1706517

**Published:** 2018-09-30

**Authors:** Chu-Yao Tseng, Ching-Wen Huang, Hsin-Chia Huang, Wei-Chen Tseng

**Affiliations:** ^1^Division of Chinese Acupuncture and Traumatology, Center of Traditional Chinese Medicine, Chang Gung Memorial Hospital, Taoyuan, Taiwan; ^2^Chang Gung University College of Medicine, Taoyuan, Taiwan; ^3^Division of Chinese Acupuncture and Traumatology, Center of Traditional Chinese Medicine, Chang Gung Memorial Hospital, Taipei, Taiwan

## Abstract

Traditional Chinese medicine (TCM) divides fracture treatment into three stages. Many TCM herbs and formulas have been used to treat fractures for thousands of years. However, research regarding the Chinese herbal products (CHPs) that should be used at different periods of treatment is still lacking. This study aims to identify the CHPs that should be used at different periods of treatment as well as confirm the TCM theory of fracture periods medicine. We used prescriptions of TCM outpatients with fracture diagnoses analyzed using the Chang Gung Research Database (CGRD) from 2000 to 2015. According to the number of days between the date of the fracture and the clinic visit date, all patients were assigned to one of three groups. Patients with a date gap of 0-13 days were assigned to the early period group; those with a date gap of 14-82 days were assigned to the middle period group; and those with a date gap of 83-182 days were assigned to the late period group. We observed the average number of herbal formulas prescribed by the TCM doctor at each visit was 2.78, and the average number of single herbs prescribed was 6.47. The top three prescriptions in the early fracture period were Zheng-gu-zi-jin-dang, Shu-jing-huo-xue-tang, and Wu-ling-san. In the middle fracture period, the top three formulas were Zheng-gu-zi-jin-dang, Shu-jing-huo-xue-tang, and Zhi-bai-di-huang-wan. In the late fracture period, the top three formulas were Shu-jing-huo-xue-tang, Gui-lu-er-xian-jiao, and Du-huo-ji-sheng-tang. The main single herbs used in the early fracture period were Yan-hu-suo, Gu-sui-bu, and Dan-shen. From the middle to the late period, the most prescribed single herbs were Xu-duan, Gu-sui-bu, and Yan-hu-suo. We concluded that the results showed that the CGRD utilization pattern roughly meets the TCM theory at different fracture periods.

## 1. Introduction

Fracture healing is the tissue repair between fracture ends. Fracture healing is a complex and continuous process that can be divided into three periods: the inflammatory phase, the reparative phase, and the remodeling phase [1–5].

The inflammatory phase is dominated by vascular events. A hematoma is formed after fracture, which provides the building blocks for healing. Macrophages, neutrophils, and platelets release several cytokines including tumor necrosis factor alpha (TNF-*α*), interleukin-1 and interleukin-6 (IL-1, -6), bone morphogenetic protein-2 (BMP-2), transforming growth factor beta (TGF-*β*), and platelet-derived growth factor (PDGF). TNF-*α*, IL-1, and IL-6 play a role in initiating the repair cascade when hematoma is formed. PDGF, BMP-2, and TGF-*β* expression increase to initiate callus formation. Those cytokines may be detected as early as 24 hours after injury. Next, reabsorption occurs at the fracture edges, which makes fracture lines radiographically different 5 to 10 days after injury. Hereafter, multipotent cells are transformed into osteoprogenitor cells, which start to form new bone. In the reparative phase, new blood vessels induced by vascular endothelial growth factor (VEGF) supply nutrients to the cartilage that forms at the fracture site. New endochondral bone creates a collar around the fractured area by callus formation. Transforming growth factor beta 2, beta 3 (TGF-*β*2, -*β*3), and growth differentiation factors-5 (GDF-5) reach peak due to their involvement in chondrogenesis and endochondral ossification process. The callus is highly cartilaginous in the early reparative phase, but endochondral calcification begins during the remodeling phase. Bone morphogenetic protein-3, protein-4, protein-7 (BMP-3, -4, -7), and BMP-8 rise with osteoclast recruitment and cartilage resorption. Clinical union of the fracture occurs in the late reparative phase. When clinical union occurs, the patient can move the injured limb without significant pain. Clinical union may occur before radiographic union, because the initial callus is cartilaginous, which does not display on radiographic images. Clinical union typically indicates the end of the reparative phase. In the remodeling phase, the endochondral callus becomes ossified completely, and the bone goes through structural remodeling. IL-1 and IL-6 rise again associated with bone remodeling coupled with osteoblast activity. The TGF-*β* family diminished expression when marrow was established. Remodeling speed depends on age. In young children, remodeling occurs quickly; young children remodel their entire skeleton every year. The rate of skeletal remodeling falls to approximately 10 percent per year by late childhood and continues at approximately this level throughout life [6, 7].

Intervention for promoting fracture healing may use parathyroid hormone (PTH) throughout all phases, anti-sclerostin or anti-Dickkopf-related protein 1 antibodies in late endochondral phase or bone remodeling phase, and BMP-2 in inflammatory phase [8].

According to traditional Chinese medicine (TCM) theory, the treatment for fracture includes three different periods. When a fresh bone fracture occurs at early period, the typical symptoms are pain and tissue swelling around the broken bone, which accord with “blood stasis” pattern and “qi stagnation” pattern. The goals of TCM principle are “quickening the blood, transforming stasis, and promoting movement of qi” to relieve pain and reduce swelling. The middle period occurs after the initial symptoms of the fracture improve, including the pain, the blood stasis, and tissue swelling. However, the site of the fracture is still weak and tender because the bone and the sinews have not yet connected. The goal of TCM principle is “to join the bone, soothe the sinews, and harmonize construction of new sinews and bone” in the middle period. The late period is characterized by the fracture initially healing, but not yet solid. At this stage, splint fixation has been lifted, and the immobilization of limbs resulted in atrophy of those muscles near fracture site. The strength and function in the fracture site have not yet been regained, which is in accord with “deficiency in both qi and blood” pattern and “liver-kidney depletion” pattern. The goal of TCM principle is to “supply qi, nourish the blood, enrich the kidney and liver, and strengthen the bone and sinew” for regaining the normal function in the affected area in the late period.

The study of the use of traditional Chinese medicine in patients with fractures in Taiwan showed that the most commonly used formula for patients with fractures was Shu-jing-huo-xue-tang, and the most commonly used single herb was Gu-sui-bu. The same study also found that the use of traditional Chinese medicine could reduce the number of fractures within six months as well as hospitalization costs [9].

The purpose of this study was to analyze the medical records of TCM patients with fracture diagnoses in TCM clinics in the Chang Gung Memorial Hospitals in Taipei, Linkou, and Taoyuan from 2000 to 2015 and to analyze the types and frequency of traditional Chinese medicine treatments in the three periods of fracture healing. This study could provide a reference for doctors' prescriptions and a clinical trial basis of bone fracture healing in the future.

## 2. Materials and Methods

### 2.1. Data Source

*Source of Participants.* We collected the medical records of outpatients with fracture diagnoses in the TCM clinics of the Chang Gung Memorial Hospitals of Taipei, Linkou, and Taoyuan from 2000 to 2015. Diagnosis codes should correspond to 800-829 in the International Classification of Disease, 9th Revision, Clinical Modification (ICD-9-CM) format for primary or secondary diagnosis by the Chang Gung Research Database. The sites of fractures were classified by ICD-9-CM codes 800-804 (fracture of the skull), 805-809 (fracture of neck and trunk), 810-819 (fracture of upper limbs), and 820-829 (fracture of lower limbs). The medical records included the following information: (1) medical record number; (2) date of visit; (3) traditional Chinese medicine type, frequency of administration, dosage, and days of medication; (4) patient complaints; (5) clinical observation; (6) diagnosis codes; and (7) outpatient visit number.

### 2.2. Study Population and Variables

Medical records were collected by the Chang Gung Research Database system. After screening, there were 1376 TCM outpatients diagnosed with fractures from 2000 to 2015 according to the clinics' medical records; patients were included in or excluded from this research based on the following criteria.

*Inclusion Criteria*

1. The date of fracture could be determined by medical record.
2. The prescribed TCM herbs, such as concentrated medicine, were reimbursed by The National Health Insurance in Taiwan.
3. There was no missing dosage, frequency, or number of days of TCM medicine.

*Exclusion Criteria*

1. The date of fracture could not be determined by medical record.
2. The prescribed TCM herbs, such as crude herbs, were not reimbursed by The National Health Insurance in Taiwan.
3. There was missing dosage, frequency, or number of days of TCM medicine.

**Figure 1:**
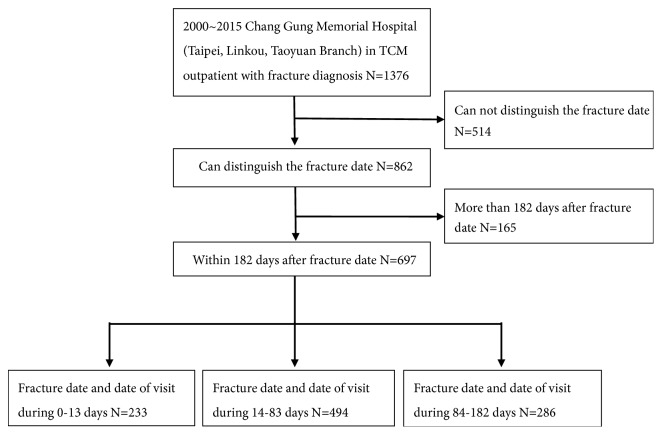
Flow recruitment chart of subjects from Chang Gung Research Database TCM outpatient with fracture diagnosis from 2000 to 2015.

According to the medical records of individual cases, a total of 862 people could determine the date of fracture, including 697 patients whose first visit was within 182 days of the date of fracture. The date of the visit minus the date of fracture resulted in the date gap that was used to assign patients to three different fracture periods. According to the TCM theory, we determined that a date gap between 0 and 13 days was assigned to the early period group, which contained 233 people. A date gap between 14 and 83 days was assigned to the middle period group, which contained 494 people. A date gap between 83 and 182 days was assigned to the late period group, which contained 286 people as shown in Figure 1.

### 2.3. Ethical Considerations

All of the data from the Chang Gung Research Database could not identify individual patients and could only be analyzed by the hospitals' computers. The Chang Gung Medical Foundation Institutional Review Board approved this research (IRB No.: 201600545B0).

### 2.4. Statistical Analysis

All statistical analyses were performed using SAS Enterprise Guide 5.1 (SAS Institute, Cary, NC). Data analysis comprised descriptive statistics, including the frequency of prescribed TCM formulas and single herbs for treating fractures. Single-factor analysis of variance (ANOVA) was used to compare age associations in different fracture period groups. A chi-square test was used to compare gender and fracture sites in different fracture period groups.

## 3. Results

From the Chang Gung Research Database, we distributed patients diagnosed with fractures on the basis of the inclusion and exclusion criteria into three groups: (1) early period of 0-13 days, (2) middle period of 14-83 days, and (3) late period of 84-182 days (Table 1). There was no significant difference between each group with respect to sex (P=0.185), age (P=0.419), or fracture site (P=0.570). We found the mean number of visits in early period (1.39±0.73) to be lower than in the middle period (3.06±2.72) and late period (3.70±3.81). The average number of herbal formula types prescribed by TCM doctors at each visit was 2.78 and the average number of single herb types was 6.47 (Table 1). We analyzed the Chinese herbal formulas prescribed by TCM doctors in each group. The most common herbal formula in the early period was Zheng-gu-zi-jin-dang (46.3%), followed by Shu-jing-huo-xue-tang (23.5%), Wu-ling-san (19.8%), and Shen-tong-zhu-yu-tang (13.9%). An additional analysis determined an average daily dose of the top eight formulas in the early period group of approximately 4.2 g to 6.4 g. The most common single herb prescribed in the early period was Yan-hu-suo (51.2%), followed by Gu-sui-bu (33.6%) and Dan-shen (30.9%). The average daily dose of the top eight single herbs was 0.8 to 1.9 g (Table 2).

The most commonly prescribed formula in the middle period was Zheng-gu-zi-jin-dang (26.6%), followed by Shu-jing-huo-xue-tang (21.5%), Zhi-bai-di-huang-wan (17.2%), and Xue-fu-zhu-yu-tang (12.9%). The average daily dose of the top eight formulas in the middle period group was approximately 3.0 g to 5.2 g. The most common single herb in middle period was Xu-duan (53.0%), followed by Gu-sui-bu (50.6%) and Yan-hu-suo (47.2%). The average daily dose of the top eight single herbs was 0.8 g to 1.4 g (Table 3).

The most common formula in the late period was Shu-jing-huo-xue-tang (21.7%), followed by Gui-lu-er-xian-jiao (15.5%), Du-huo-ji-sheng-tang (12.9%), and Zhi-bai-di-huang-wan (12.7%). The average daily dose of the top eight formulas in the late period group was approximately 3.1 g to 6.0 g. The most common single herb in the late period was Xu-duan (45.3%), followed by Gu-sui-bu(39.1%) and Yan-hu-suo (37.0%). The average daily dose of the top eight single herbs was 0.8 g to 1.5 g (Table 4).

We summarized TCM indication of the common herbal formulas and single herbs in Table 5 and the ingredient herbs of herbal formulas and single herbs with scientific name mentioned in the study were listed in Table 6.

## 4. Discussion

We determined that the average number of herb types used as medical treatment for fracture outpatients was 6.47 single herbs and 2.78 formulas (Table 1). The results showed that multiple TCM formulas and herbs were used to treat fracture outpatients in the Chang Gung Memorial Hospital. The use of multiple formulas may be due to traditional Chinese medicines used by TCM doctors in Taiwan being concentrated herbal extracts. Each formula is made into a concentrated powder that is easy to mix with other formulas and easy to package. The use of high-frequency formulas and herbs might be the basis of fracture treatment, and the remaining herbs could be chosen according to the patient's condition.

From the early to the middle period, the two most common formulas were Zheng-gu-zi-jin-dang and Shu-jing-huo-xue-tang. Zheng-gu-zi-jin-dang was most commonly used in fractures where injuries were due to knocks and falls, sinew damage, or bone fracture. The possible pharmacological mechanisms are antimicrobial [10] and gastroprotective effects [11].

Shu-jing-huo-xue-tang was prescribed for rheumatism and venous stasis, but it was also used as a formula for general bone pain and for patients with fractures. It could reduce pain and blood stasis and played a role in antiapoptosis [12] and neuroprotection [13]. The above two formulas occupied a high proportion of the treatments for each fracture period and could be used as the basis of fracture treatment and combined with other formulas. Zheng-gu-zi-jin-dang was prescribed for nearly half of the patients in the early period, showing that the tendency of doctors to use Zheng-gu-zi-jin-dang is consistent in the early stage of fractures. It is presumed that the patients suffered from severe bruising and pain due to fractures in the early stage, so drugs were used to relieve blood stasis and pain. The use of Zheng-gu-zi-jin-dang is consistent with the principle of traditional Chinese medicine theory of fracture treatment. More than one-fifth of the patients in each stage were prescribed Shu-jing-huo-xue-tang; in traditional Chinese medicine, it is often used in pain diseases such as arthritis. It is deduced that a high proportion of patients use Shu-jing-huo-xue-tang for fractures as a drug to control pain and to be used as a base with other prescriptions.

In addition, in the early fracture period, other common formulas are used for blood-quickening, stasis-transforming, and pain relief, such as Si-wu-Tang, Shen-tong-zhu-yu-tang, and Xue-fu-zhu-yu-tang. For the initial swelling of the fracture, formulas, such as Wu-ling-san, are used to disinhibit water and disperse swelling by increasing urinary output [14]. The pharmacological mechanisms of Xue-fu-zhu-yu-tang are due to its proangiogenic [15] and neuroprotective effects [13]. The results could be consistent with the TCM theory of “quickening the blood and transform stasis and promoting movement of qi” in the early fracture period.

In middle fracture period, formulas similar to the formulas used in the early period were used, with the exception of Dang-gui-nian-tong-tang and Shao-yao-gan-cao-tang. Dang-gui-nian-tong-tang has commonly been used to treat swelling and tenderness of joints [16]. Shao-yao-gan-cao-tang was used for reducing limb or abdomen pain. The possible pharmacological mechanism is anti-inflammation [17]. According to the results, treatment in the middle period met the TCM goal of “joining the bone, soothing the sinews, and harmonizing construction of new sinews and bone”.

In the late fracture period, we found that Gui-lu-er-xian-jiao and Du-huo-ji-sheng-tang were used to supplement liver and bone. Gui-lu-er-xian-jiao contained BMP-4 which could promote the growth of bone [18] and supply kidney yin and yang. Du-huo-ji-sheng-tang could delay the aging process [19] and decrease the severity of inflammation [20]. The results could meet the TCM goals to “supplement qi, nourish the blood, enrich the kidney and liver, and strengthen the bone and sinew” in the late period.

The results demonstrated that different fracture periods used different single herbs. The most common single herb in the early period was Yan-hu-suo, which was prescribed for more than 50% of outpatients. Yan-hu-suo's medical effects are quickening the blood and moving qi, and it also plays a role in anti-inflammation and pain relief [21] and reduced tissue swelling. Gu-sui-bu's medical effects are quickening the blood, strengthening sinew and bone, supporting the kidneys, and promoting osteoclast proliferation [22]. Dan-shen's medical effects are quickening the blood, transforming stasis, and perhaps accelerating early-stage fracture healing [23]. The above three single herbs corresponded with the treatment principle of dispersed stasis and reduced swelling in the early fracture period.

From the middle to late fracture periods, the top three single herbs were Xu-duan, Gu-sui-bu, and Yan-hu-suo. Xu-duan and Gu-sui-bu showed a similar prescribed proportion of outpatients. It could be concluded that the doctor prescribing two single herbs at the same time was quite common. Yan-hu-suo was also prescribed a lot in the middle and late fracture periods, and its medical effects were moving qi and relieving pain.

In the other single herbs used regularly in the early and middle fracture periods, we could see a variety of blood-quickening, stasis-transforming herbs, such as Chuan-niu-xi, Hu-zhang, Chi-shao, and Dan-shen. This showed that, in the early and middle fracture periods, injured tissue was still in a state of stasis and swollen, and it was necessary to promote blood circulation and harmonize construction with blood-quickening, stasis-transforming single herbs of which there were a variety from which to choose. Clinical doctors prescribed herbs similar to those used to strengthen sinew and bones, such as Xu-duan and Gu-sui-bu. According to the different patients' conditions, the doctors choose other herbs, such as blood-quickening stasis-transforming herbs, so that the average number of single herbs prescribed by the doctor reached 6.47 per person.

In previous studies based on the National Health Insurance Research Database (NHIRD) Liao et al. showed the top ten commonly prescribed formulas for treating fractures were Shu-jing-huo-xue-tang, Zheng-gu-zi-jin-dang, Shao-yao-gan-cao-tang, Xue-fu-zhu-yu-tang, Dang-gui-nian-tong-tang, Du-huo-ji-sheng-tang, Zuo-gui-wan, Shen-tong-zhu-yu-tang, Liu-wei-di-huang-wan, and Fu-yuan-huo-xue-tang [9]. Seven formulas and five single herbs of the study also appeared in this study, confirming that the Chang Gung Research Database is highly correlated with the Taiwan Health Insurance Research Database (NHIRD) [24]. In both studies, similar results showed that the daily usage of formulas was greater than the daily usage of single herbs. Therefore, TCM doctors in Taiwan mainly use formulas and use single herbs as auxiliary. However, the previous study did not mention that prescriptions might be altered with a different period of fracture. This study was based on the statistics of patients of TCM department in CGMH within half a year after the fracture, which excluded most of the patients who have been healed and continue to receive TCM treatment. The medical records of the fracture diagnosis code recorded by the TCM doctor can confirm that the patient is fractured and the relevant drugs have been prescribed. In the results, we found that the top three formulas of each fracture stage account for 12.9% ~ 46.3%, and the top three single herbs are 30.9% ~ 53% higher than the previous studies, indicating that these drugs are more commonly used for the treatment of fractures by doctors in CGMH TCM comparing to the other doctors in TCM in Taiwan.

On the other hand, Gui-lu-er-xian-jiao was listed in the top three commonly used formulas for treating late period fracture in the CGMH Chinese medicine department but was absent in Dr. Liao's study [9]. The main reason might be that the usage rate of Gui-lu-er-xian-jiao in other hospitals or health facilities in Taiwan was lower than in the CGMH Chinese medicine department. In both studies, Gu-sui-bu, Xu-duan, and Yan-hu-suo were on the list of most commonly used single herbs. The results revealed that the preferences for prescriptions for patients with fractures in the Chang Gung Memorial Hospital were similar to other medical facilities.

Molecular mechanisms studies of various herbs for treating fractures or promoting fracture healing are still rare today. The result of TCM formulas or single herbs in this study was based on the TCM theory. It needs more research to know what role dose these drugs play in the mechanism of fracture healing. These formulas and single herbs also provide direction for future research.

## 5. Limitations

The samples in this study came from the Chang Gung Research Database which is not a national database. The number of samples is not extensive enough, which may cause a statistical bias. In addition, the date of fracture could not be identified in approximately one-third of the samples in the study. It is unclear whether these excluded cases would affect the statistical results.

## 6. Conclusion

In the CGMH, the average number of herb types prescribed for fracture outpatients was 6.47 single herbs and 2.78 formulas. The top three prescriptions given in the early fracture period were Zheng-gu-zi-jin-dang, Shu-jing-huo-xue-tang, and Wu-ling-san as well as other types of blood-quickening stasis-transforming formulas. In the middle fracture period, the top three formulas were Zheng-gu-zi-jin-dang, Shu-jing-huo-xue-tang, and Zhi-bai-di-huang-wan. In the late fracture period, the top three formulas were Shu-jing-huo-xue-tang, Gui-lu-er-xian-jiao, and Du-huo-ji-sheng-tang. The single herbs used in the early fracture period were Yan-hu-suo, Gu-sui-bu, and Dan-shen. From the middle to late period, the most prescribed single herbs were Xu-duan, Gu-sui-bu, and Yan-hu-suo. This study showed a prescription trend in the CGMH TCM doctors for clinical fracture outpatients. The results showed a utilization pattern that roughly aligned the TCM theories at different fracture periods.

## Figures and Tables

**Table 1:** Database of fracture period group.

	Early period	Middle period	Late period	P value
Number of sex				0.185
Male	106	218	130	
Female	127	276	156	
Age ±SD	48.10±18.28	48.65±16.74	49.97±16.45	0.419

No. of case fracture site				

Skull and neck	3	6	6	0.570
Trunk	42	110	63	
Upper limb	107	213	111	
Lower limb	81	165	106	

Number of visit ±SD	1.39±0.73	3.06±2.72	3.70±3.81	N/A

Average numbers of herbal products: formulas were 2.78 types and single herbs were 6.47 types prescription by TCM doctor in each visit.

**Table 2:** Early fracture period (0-13 days).

Herb formulas	Frequency of prescription N (%)	Average daily dose (g) ±SD	Single herb	Frequency of prescription N (%)	Average daily dose (g) ±SD
Zheng-gu-zi-jin-dang	150(46.3)	5.5±2.13	Yan-hu-suo	166(51.2)	1.4±0.53
Shu-jing-huo-xue-tang	76(23.5)	6.4±1.67	Gu-sui-bu	109(33.6)	1.5±0.67
Wu-ling-san	64(19.8)	6.2±1.63	Dan-shen	100(30.9)	1.6±0.43
Shen-tong-zhu-yu-tang	45(13.9)	4.5±1.45	Xu-duan	75(23.1)	1.3±0.74
Xue-fu-zhu-yu-tang	44(13.6)	5.8±2.06	Ze-lan	51(15.7)	1.7±0.91
Long-dan-xie-gan-tang	40(12.3)	4.2±1.20	Hu-zhang	50(15.4)	1.9±0.95
Zhi-bai-di-huang-wan	39(12.0)	4.5±1.10	Sang-zhi	47(14.5)	1.0±0.24
Si-wu-tang	37(11.4)	4.5±1.58	Hong-hua	43(13.3)	0.8±0.16

**Table 3:** Middle fracture period (14-83 days).

Herb formulas	Frequency of prescription N (%)	Average daily dose (g) ±SD	Single herb	Frequency of prescription N (%)	Average daily dose (g) ±SD
Zheng-gu-zi-jin-dang	403(26.6)	4.7±2.56	Xu-duan	804(53.0)	1.3 ±0.64
Shu-jing-huo-xue-tang	326(21.5)	5.1±1.98	Gu-sui-bu	767(50.6)	1.4 ±0.65
Zhi-bai-di-huang-wan	260(17.2)	5.1±1.89	Yan-hu-suo	716(47.2)	1.2 ±0.48
Xue-fu-zhu-yu-tang	195(12.9)	5.1±2.09	Dan-shen	345(22.8)	1.4 ±0.47
Dang-gui-nian-tong-tang	175(11.5)	4.6±2.47	Chi-shao	227(15.0)	0.8 ±0.28
Shen-tong-zhu-yu-tang	155(10.2)	4.1±1.57	Chuan-niu-xi	219(14.4)	1.0 ±0.43
Shao-yao-gan-cao-tang	151(10.0)	3.0±1.11	Sang-zhi	189(12.5)	1.0 ±0.58
Du-huo-ji-sheng-tang	147(9.7)	5.2±1.91	Gui-jia	176(11.6)	0.9 ±0.31

**Table 4:** Late fracture period (84-182 days).

Herb formulas	Frequency of prescription N (%)	Average daily dose (g) ±SD	Single herb	Frequency of prescription N (%)	Average daily dose (g) ±SD
Shu-jing-huo-xue-tang	230(21.7)	4.2±1.55	Xu-duan	480(45.3)	1.5±0.78
Gui-lu-er-xian-jiao	164(15.5)	4.3±1.96 (PC)*∗*	Gu-sui-bu	414(39.1)	1.5±0.77
Du-huo-ji-sheng-tang	137(12.9)	5.4±2.05	Yan-hu-suo	392(37.0)	1.1±0.38
Zhi-bai-di-huang-wan	134(12.7)	6.0±2.60	Dan-shen	176(16.6)	1.2±0.43
Zheng-gu-zi-jin-dang	132(12.5)	3.7±2.37	Chi-shao	149(14.1)	0.9±0.21
Shao-yao-gan-cao-tang	128(12.1)	3.1±1.55	Chuan-niu-xi	141(13.3)	0.9±0.38
Xue-fu-zhu-yu-tang	121(11.4)	3.6±1.76	Gui-jia	140(13.2)	0.9±0.26
Dang-gui-nian-tong-tang	101(9.5)	4.0±1.43	Chen-pi	117(11.1)	0.8±0.11

*∗* Formulation of Gui-lu-er-xian-jiao was pill whose weight was 0.5 gram per piece.

**Table 5:** TCM indication of the common herbal formulas and single herbs.

**TCM formulas**	Indications in TCM use
Zheng-gu-zi-jin-dang(Bone-Righting Purple Gold Elixir)	Damage to sinew and bone especially fractures
Shu-jing-huo-xue-tang(Channel-Coursing Blood-Quickening Decoction)	Wind-cold-damp impediment with blood deficiency and blood stasis
Wu-ling-san(Poria Five Powder)	Water-damp collecting internally and regulating water metabolism to reduce swelling
Gui-lu-er-xian-jiao(Tortoise Shell and Deerhorn Two Immortals Glue)	Kidney depletion or yin and yang vacuity, impotence premature ejaculation
Zhi-bai-di-huang-wan(Anemarrhena, Phellodendron, and Rehmannia Pill)	Effulgent yin vacuity fire, steaming bone tidal heat, vacuity vexation, back pain.
Du-huo-ji-sheng-tang(Pubescent Angelica and Mistletoe Decoction)	Dual vacuity of liver and kidney, insufficiency of qi and blood, low back and knee pain
**Single herbs**	

Xu-duan	Enrich the liver and kidney, strengthen sinew and bone, pain and swelling in the limbs
Gu-sui-bu	Mending soft tissues, bones and tendons due to falls, fractures, sprains and contusions
Yan-hu-suo	Any type of pain affecting all limb and truck due to contusion, blood stagnation, and Qi stagnation.
Dan-shen	Quicken the blood and transform stasis

**Table 6:** The ingredient herbs contained in the common herbal formulas treating bone fracture.

Herbal formulas (number of herbs)	Ingredient herbs
Zheng-gu-zi-jin-dang(12)	Flos Caryophylli (Ding-xiang), Radix Aucklandiae (Mu-xiang), Pasta Acaciae seu Uncariae (Ercha), Sanguis Draconis (Xue-jie), Radix et Rhizoma Rhei (Da-huang), Flos Carthami (Hong-hua), Radix Angelicae Sinensis (Dang-gui), Sclerotum Poriae Cocos (Fu-ling), Nelumbo nucifera Gaertn (Lian-rou), Radix Paeoniae Alba (Bai-shao), Radix Glycyrrhizae (Gan-cao), Cortex Moutan (Mu-dan-pi)

Shu-jing-huo-xue-tang(16)	Radix Angelicae Sinensis (Dang-gui), Radix Glycyrrhizae (Gan-cao), Radix Paeoniae Alba (Bai-shao), Rhizoma Atractylodis (Cang-zhu), Radix Rehmanniae (Sheng-di-huang), Radix Cyathulae (Chuan-niu-xi), Pericarpium Citri Reticulatae (Chen-pi), Radix Clematidis (Wei-ling-xian), Semen Persicae (Tao-ren), Rhizoma Chuanxiong (Chuan-xiong), Radix et Rhizoma Notopterygii (Qiang-huo), Radix Stephaniae Tetrandrae (Han-fang-ji), Radix Angelicae Dahuricae (Bai-zhi), Radix Gentianae (Long-dan-cao), Sclerotum Poriae Cocos (Fu-ling), Rhizoma Zingiberis Recens (Sheng-jiang)

Wu-ling-san(5)	Rhizoma Alismatis (Ze-xie), Sclerotum Poriae Cocos (Fu-ling), Polyporus (Zhu-ling), Rhizoma Atractylodis Macrocephalae (Bai-zhu), Ramulus Cinnamomi(Gui-zhi)

Shen-tong-zhu-yu-tang(12)	Radix Gentianae Macrophyllae (Qin-jiao), Rhizoma Chuanxiong (Chuan-xiong), Flos Carthami (Hong-hua), Semen Persicae (Tao-ren), Radix Glycyrrhizae (Gan-cao), Radix et Rhizoma Notopterygii (Qiang-huo), Radix Angelicae Sinensis (Dang-gui), Resina Commiphora (Mo-yao), Feces Trogopterori (Wu-ling-zhi), Rhizoma Cyperi (Xiang-fu), Radix Cyathulae (Chuan-niu-xi), Pheretima (Di-long)

Xue-fu-zhu-yu-tang(11)	Radix Angelicae Sinensis (Dang-gui), Radix Rehmanniae (Sheng-di-huang), Flos Carthami (Hong-hua), Semen Persicae (Tao-ren), Fructus Aurantii (Zhi-ke), Radix Paeoniae Rubra (Chi-shao), Radix Bupleuri (Chai-hu), Radix Glycyrrhizae (Gan-cao), Rhizoma Chuanxiong (Chuan-xiong), Radix Platycodi (Jie-geng), Radix Cyathulae (Chuan-niu-xi)

Long-dan-xie-gan-tang(10)	Radix Gentianae(Long-dan-cao), Radix Scutellariae (Huang-qin), Fructus Gardeniae(Zhi-Zi), Caulis Akebiae(Mu-tong), Semen Plantaginis(Che-qian-zi), Rhizoma Alismatis (Ze-xie), Radix Bupleuri (Chai-hu), Radix Rehmanniae (Sheng-di-huang), Radix Angelicae Sinensis (Dang-gui), Radix Glycyrrhizae (Gan-cao)

Zhi-bai-di-huang-wan(8)	Rhizoma Anemarrhenae (Zhi-mu), Cortex Phellodendri(Huang-bo),Radix Rehmanniae Preparata (Shu-di-huang), Fructus Corni (Shan-zhu-yu), Rhizoma Dioscoreae (Shan-yao), Rhizoma Alismatis (Ze-xie), Sclerotum Poriae Cocos (Fu-ling), Cortex Moutan (Mu-dan-pi)

Si-wu-tang(4)	Radix Angelicae Sinensis (Dang-gui), Rhizoma Chuanxiong (Chuan-xiong), Radix Paeoniae Alba (Bai-shao), Radix Rehmanniae Preparata (Shu-di-huang)

Dang-gui-nian-tong-tang(14)	Herba Artemisiae Scopariae (Yin-chen), Radix et Rhizoma Notopterygii (Qiang-huo), Radix Saposhnikoviae (Fang-feng), Rhizoma Cimicifugae (Sheng-ma), Radix Puerariae (Ge-gen), Rhizoma Atractylodis Macrocephalae (Bai-zhu), Rhizoma Atractylodis (Cang-zhu), Radix Glycyrrhizae (Gan-cao), Radix Scutellariae (Huang-qin), Rhizoma Anemarrhenae (Zhi-mu), Radix Sophorae Flavescentis (Ku-shen), Radix Angelicae Sinensis (Dang-gui), Polyporus (Zhu-ling), Rhizoma Alismatis (Ze-xie)

Shao-yao-gan-cao-tang(2)	Radix Paeoniae Alba (Bai-shao), Radix Glycyrrhizae (Gan-cao)

Du-huo-ji-sheng-tang(15)	Radix Angelicae Pubescentis (Du-huo), Herba Taxilli (Sang-ji-sheng), Cortex Eucommiae (Du-zhong), Radix Cyathulae (Chuan-niu-xi), Radix et Rhizoma Asari (Xi-xin), Radix Gentianae Macrophyllae (Qin-jiao), Cortex Cinnamomi (Rou-gui), Sclerotum Poriae Cocos (Fu- ling), Radix Saposhnikoviae (Fang-feng), Rhizoma Chuanxiong (Chuan-xiong), Radix Ginseng (Ren-shen), Radix Angelicae, Radix Glycyrrhizae (Gan-cao) Sinensis (Dang-gui), Radix Paeoniae Alba (Bai-shao), Radix Rehmanniae Preparata (Shu-di-huang)

Gui-lu-er-xian-jiao(4)	Mature Cornu Cervi Pantotrichum(Lu-jiao), Plastrum Testudinis(Gui-ban), Fructus Lycii(Gou-qi-zi), Radix Ginseng (Ren-shen)

Single herbs with scientific name mentioned in the study: Yan-hu-suo (Rhizoma Corydalis), Gu-sui-bu (Rhizoma Drynariae), Dan-shen (Radix Salviae Miltiorrhizae), Xu-duan (Radix Dipsaci), Ze-lan (Herba Lycopi), Hu-zhang (Rhizoma Polygoni Cuspidati), Sang-zhi (Ramulus Mori), Hong-hua (Flos Carthami), Chi-shao (Radix Paeoniae Rubra), Chuan-niu-xi (Radix Cyathulae), Gui-jia (Plastrum Testudinis), Chen-pi (Pericarpium Citri Reticulatae).

## Data Availability

Data are available from Chang Gung Research Database provided by Chang Gung Memorial Hospital. Due to legal restrictions imposed by the Taiwan government with respect to the “Personal Information Protection Act”, data cannot be made in public. Data requests as a formal proposal can be sent to Chang Gung Memorial Hospital (https://www.cgmh.org.tw/).
